# Supplementary figures and images for: Image segmentation of cervical grainy sandy patches lesions associated with female genital schistosomiasis using deep convolutional neural network with U-NET architecture
Source: PLoS Negl Trop Dis. 2026 Mar 5;20(3):e0014037. doi: 10.1371/journal.pntd.0014037 (PMC12981554; doi:10.1371/journal.pntd.0014037)

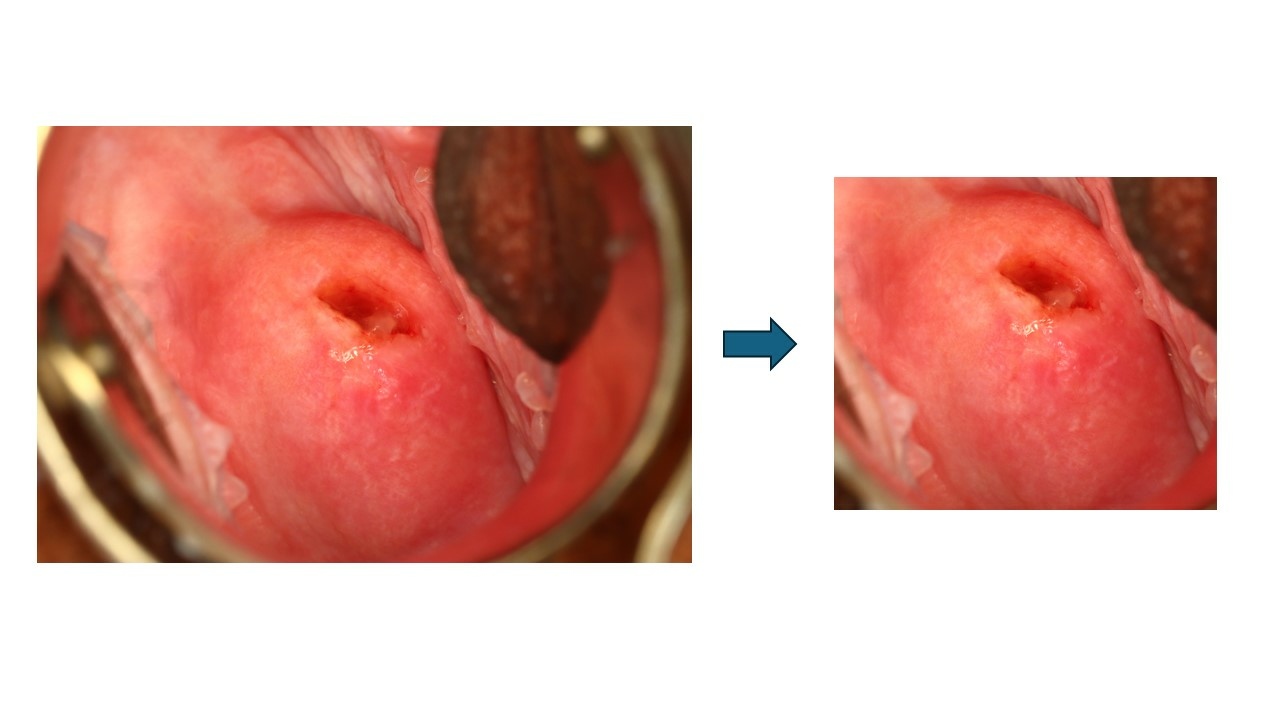

Supplement: S1 Fig — The annotation image is aligned with the original, unannotated image. To perform image differencing, both images must be the same size and registered in the same coordinate space. Registration is achieved by matching Scaled Invariant Feature Transforms and using Random Sample Consensus to find the best transformation matrix between the two images. The original image is then cropped to match the dimensions of the cropped annotation. Created using source images from [19]. (TIF) [file pntd.0014037.s001.tif]

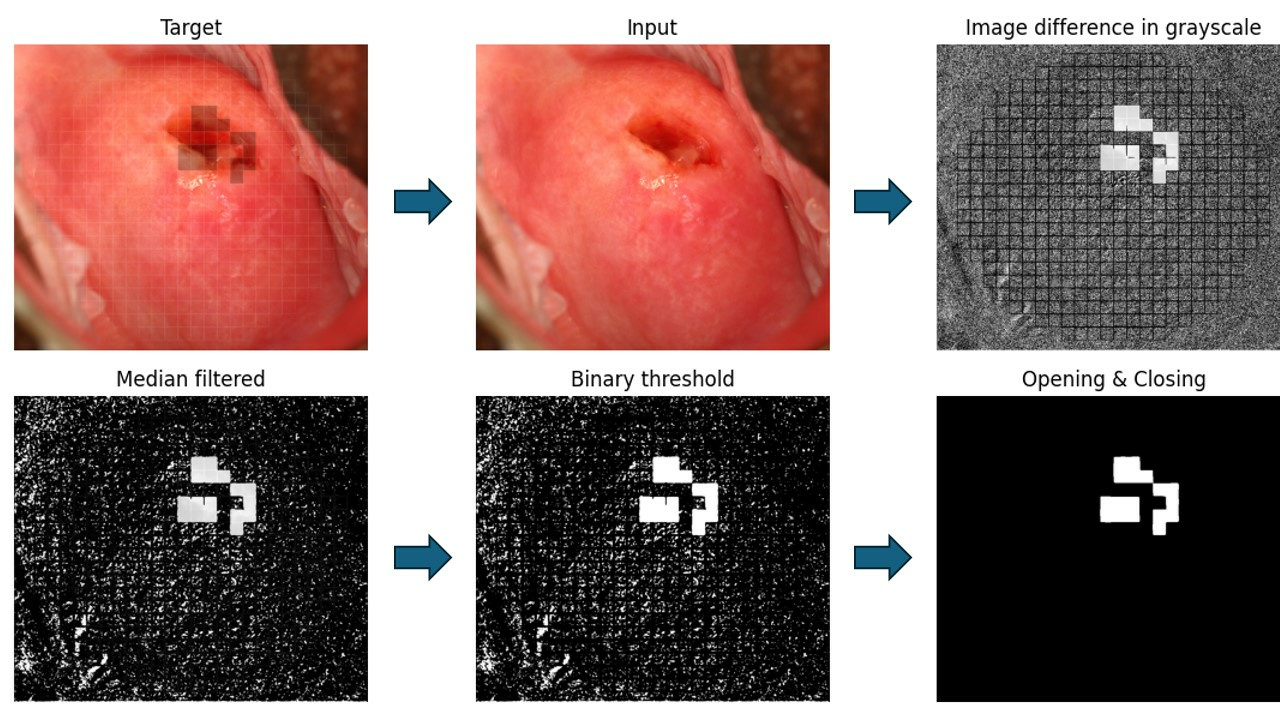

Supplement: S2 Fig — The annotation mask is extracted by computing the pixel-wise difference between them to isolate annotated regions. The difference image is converted to grayscale and filtered with a median filter to reduce noise. Afterward, binarization at a threshold of 0.5 creates a binary mask, and morphological opening and closing operations are applied to remove residual noise and fill small gaps. The resulting mask is a clean binary representation of the annotations. Created using source images from [19]. (TIF) [file pntd.0014037.s002.tif]
