# Supplementary material for: Image segmentation of cervical grainy sandy patches lesions associated with female genital schistosomiasis using deep convolutional neural network with U-NET architecture
Source: PLoS Negl Trop Dis. 2026 Mar 5;20(3):e0014037. doi: 10.1371/journal.pntd.0014037 (PMC12981554; doi:10.1371/journal.pntd.0014037)
Supplement: S1 Appendix — (DOCX) [file pntd.0014037.s003.docx]

**Specular reflection methodology**

SRs are characterized by high-intensity pixels and regions with steep-intensity gradients. Two detection methods leverage these characteristics: First, analyzing RGB channel intensities, high values in all three channels suggest SRs. Given that cervical images naturally have a prominent red channel because of the tissue's red/pinkish coloration [23], attention was directed to the green and blue channels, whose differences suggest SRs. Second, image gradients are calculated to identify areas with sharp intensity changes. Combining these methods helps ensure accurate SR detection.

Image contrast was first increased by applying morphological operations to enhance feature visibility. The *tophat* [24] operation is added to the original image, and the *blackhat* [24] operation is subtracted. Both are applied using a 5x5 elliptical kernel. To set an adaptable threshold, a dynamic calculation, adapted from Nie et al. [21], is used (Eq. S1):

$threshold=\max_{gb}-\tau\cdot\frac{\sigma_{g}+\sigma_{b}}{2}$ (S1)

Where max_gb_ is the maximum green and blue channel intensity, σ_g_ and σ_b_ are their standard deviations, and τ (set to 2 based on empirical testing) tunes the threshold. This threshold is applied to each image, creating a mask where the green and blue channels exceed it.

In addition, a gradient-based mask is created to detect regions of high relative intensity change. After converting the enhanced contrast image to grayscale, gradients in both the x and y directions are calculated and combined. This combined gradient map is thresholded at an intensity of 80. The final mask, combining absolute (channel-based) and relative (gradient-based) highlights, is ready for inpainting.

**Hyper-parameter tuning from previous experimentation**

For hyper-parameter tuning, grid search was employed, focusing on the Adam optimizer's learning rate and the focal loss function's alpha and gamma values.

These parameters were tuned because they significantly impact model performance, particularly in cases of class imbalance, as seen in our dataset. The search space for each parameter is detailed in S1 Table.  **S1 Table. Hyperparameter Search Space**

The batch size was fixed at 32, and the number of epochs was set to 100. These choices were influenced by the limited size of the dataset and the augmentations applied to balance the data and improve generalization.

After running the grid search, the model with the highest average intersection over union, a calculation representing the model's overlapping capability, across all epochs, was selected as the best-performing model. Once the model was selected, the validation loss for each epoch was analyzed, and the epoch with the lowest validation loss was selected as the model with the best generalization.
