# Supplementary material for: Image segmentation of cervical grainy sandy patches lesions associated with female genital schistosomiasis using deep convolutional neural network with U-NET architecture
Source: PLoS Negl Trop Dis. 2026 Mar 5;20(3):e0014037. doi: 10.1371/journal.pntd.0014037 (PMC12981554; doi:10.1371/journal.pntd.0014037)
Supplement: S1 Table — The batch size was fixed at 32, and the number of epochs was set to 100. These choices were influenced by the limited size of the dataset and the augmentations applied to balance the data and improve generalization. After running the grid search, the model with the highest average intersection over union, a calculation representing the model’s overlapping capability, across all epochs, was selected as the best-performing model. Once the model was selected, the validation loss for each epoch was analyzed, and the epoch with the lowest validation loss was selected as the model with the best generalization. (DOCX) [file pntd.0014037.s004.docx]

**S1 Table. Hyperparameter Search Space.**

| **Parameter** | **Search Space** |
| --- | --- |
| Learning Rate | [0.001, 0.0001] |
| Alpha (Focal Loss) | [0.1, 0.25] |
| Gamma (Focal Loss) | [2,3,5] |
