# Supplementary material for: Image segmentation of cervical grainy sandy patches lesions associated with female genital schistosomiasis using deep convolutional neural network with U-NET architecture
Source: PLoS Negl Trop Dis. 2026 Mar 5;20(3):e0014037. doi: 10.1371/journal.pntd.0014037 (PMC12981554; doi:10.1371/journal.pntd.0014037)
Supplement: Attachment — Submitted filename: Joeker et al peer review.docx [file pntd.0014037.s005.docx]

This is a well-structured paper on the development of a model to segment grainy sandy patches, with the idea of automated detection of FGS. The performance of this model is encouraging.

**General comments:**

This model focuses on one of the four FGS lesion types. This is likely to be a significant limitation of the model in terms of its diagnostic usefulness for FGS. GSPs are not often the most common lesion type reported in the literature. It is understandable why the authors may have selected GSP as a starting point for the model but this needs to be explained, with this key limitation explained early. Careful distinction is needed when discussing FGS lesions in general, not GSP specifically.

There needs to be more explanation of why a segmentation model was chosen over a classification model. How do the authors foresee a segmentation model being used in screening/diagnosis?

There are multiple mentions of integration into smartphones/mobile phones. L78-82 suggest that this is the only potential application of this work. However, the types of smartphones that are available and affordable in endemic low-resource settings may not always have the magnification and imaging capabilities to detect GSP lesions. I would encourage the authors to consider that there may be other ways of integrating this technology.

What steps were taken to avoid overfitting? As this is a relatively small dataset, some comments on how the risk of overfitting were addressed would be appropriate. The relatively small testing set (n = 87) should also be addressed when discussing the results.

More information on the types of lesions seen in the dataset is warranted. The abstract states that all 583 cervical images exhibited FGS-associated lesions. Please could the authors clarify whether this was any lesion type or GSP specifically? How many images had other lesion types and did the model ever segment these lesions instead?

More information is needed on the level of imbalance in the dataset. GSP generally only take up a tiny percentage of the overall ROI. This is important for assessing class imbalance and for understanding the use of a focal loss function (and the degree of alpha and gamma used).

I cannot see any mention of ethics in this paper. It may fall under the larger study by Arenholt et al. but this needs to be clarified. Some information on how the images were handled (including data security etc) would also be beneficial.

**I would appreciate if the authors could address the following specific points:**

Abstract: Please could the authors clarify what they mean by non-invasive? Even with a smartphone, the procedure would still require a speculum exam, which many women consider highly invasive. This is also mentioned in L296 and in the conclusion. Visual diagnostics, by their nature, will always be invasive.

Abstract: The use of the term “distinct mucosal lesions” would suggest that they are distinctive from other cervical mucosal changes, which is often not the case. Suggest rewording. Mention of ‘confounding cervical diseases’ (diseases that appear visually similar to FGS) would also be beneficial.

L15-17: Diagnostic capacity is also constrained by the high levels of subjectivity. I would suggest adding this into your introduction, as reducing subjectivity is a key factor in the benefits of computer vision.

L27: Sigve’s work is not recent. This paper was published ten years ago. Since then, there have been significant advancements computer vision (and FGS research in general).

L69-76: Again, I would be hesitant to use the term “visually distinct”. There is limited evidence that GSP could be considered pathognomonic (e.g. Kjetland et al.,2005) but in another paper of hers that year there was no association with eggs found in biopsy etc.

L83-108: Some more detail on the imaging, annotation and classification process is needed. These details are essential when considering how successfully a model may be able to perform and generalise.

L91: Some brief details on the ‘standardized manner’ would be beneficial.

L96: Please could the authors comment on how many images were discarded due to ‘inadequate image quality? These were separate from the 7 lost during preprocessing? What was considered poor quality (e.g. just blurring? ROI not in view? Discharge in view?)?

L99-108: Please could the authors comment on the number of reviewers, their qualifications and training to label images? Or specifically refer the reader to the relevant paper? These sorts of details are useful when determining the accuracy of the ground truth annotations.

L98-108: How was the region of interest defined? If your model only focuses on the cervix, and not the surrounding tissue, then this will need to be specified.

L119-153: The section detailing the removal of SRs has a large amount of detail compared to other methods sections that may be more relevant to the objective of the paper (and of more interest to a PLOSNTD audience). Much of the detail could be added to a supplementary file to free up space.

L126-127 &135-136: These lines are almost identical. Suggest rewording or removing one for brevity.

L151-152: Some more information about the kinds of anomalies and outliers would be useful.

L166-167: The author could provide more detail on the type of hardware used so the reader could appreciate the limitations fully. Information on how data was secured would also be appropriate.

L169-170: Your data was longitudinal, with multiple images per woman. Please can the authors comment on how they ensured that images from one woman were not used across both training and testing datasets, as this may create an uplift in model performance. Furthermore, do the authors foresee any risk of bias by having multiple timepoints per woman? Perhaps a comment (and the small amount of evidence we have on lesion progression) on the likelihood of lesions changing over the study period might be warranted?

L238-239: The mention of visual variability and subtlety here is good. However, think should be mentioned earlier, before the discussion.

L267-284: The model has not been run using any external datasets (understandably), which needs to be mentioned. Furthermore, it does not appear that the model has been run on images that do not have FGS lesions? Without negative controls, how do we know that the model won’t segment ‘GSP-like’ areas in FGS-negative images?
