# Supplementary material for: Image segmentation of cervical grainy sandy patches lesions associated with female genital schistosomiasis using deep convolutional neural network with U-NET architecture
Source: PLoS Negl Trop Dis. 2026 Mar 5;20(3):e0014037. doi: 10.1371/journal.pntd.0014037 (PMC12981554; doi:10.1371/journal.pntd.0014037)
Supplement: Attachment — Submitted filename: Response to reviewers.docx [file pntd.0014037.s006.docx]

Response to reviewers

Manuscript ID: PNTD-D-25-00981

Title: Image segmentation of cervical grainy sandy patches lesions associated with female genital schistosomiasis using deep convolutional neural network with U-NET architecture

We thank the Editor and the reviewers for their careful evaluation of our manuscript and for their constructive comments. We have revised the manuscript accordingly and believe that these changes have significantly improved the quality and clarity of the work. Below, we provide a point-by-point response to all comments. The comment is marked with bold text, and the following text is the response. First, the Editor comments are addressed, thereafter, reviewer general comments are addressed, followed by specific comments. Changes in the manuscript are indicated by page and line numbers.

**Editor comments:**

**Comment 1: Abstract –no mention how the images were compared/classified as FGS positive.**

**Response**: Information about how the models were evaluated and which image data was compared has now been added to the abstract (page 2, lines 31-32).

**Comment 2: Introduction – while the description of similar models in use for cervical cancer sets the scene and utility of these methods for FGS, the introduction currently reads more like a review. Specific context is needed regarding FGS and the current diagnostic methods, including sensitivity and specificity.**

**Response**: The introduction has been revised to reduce emphasis on cervical cancer studies and to strengthen the focus on FGS, including current diagnostic approaches and their reported limitations in sensitivity and specificity (pages 4–5).

**Comment 3: Line 92 – given the loss of images from inadequate image quality, can the authors provide further details regarding what is meant by ‘standarized manner using a digital camera’? What was the protocol to ensure comparable images?**

**Response**: Thank you for this comment. The dataset section has now been updated to include more specific details of the image capturing procedure (page 7, lines 147-154).

**Comment 4: Line 99 – what level of image resolution is used for this the QubiFier? How do the FGS experts grade the lesions – this information could be included in the introduction?**

**Response**: These are important aspects of the annotation process and we have decided to provide more detail in the “Annotation with QubiFier” section. Additional information of the annotators and image resolution has been added on page 8 in lines 175-178. Also, a reference for additional information has been added on line 182.

**Reviewer General Comments**

**Comment 5: This model focuses on one of the four FGS lesion types. This is likely to be a significant limitation of the model in terms of its diagnostic usefulness for FGS. GSPs are not often the most common lesion type reported in the literature. It is understandable why the authors may have selected GSP as a starting point for the model but this needs to be explained, with this key limitation explained early. Careful distinction is needed when discussing FGS lesions in general, not GSP specifically.**

**Response**: Thank you for the comment. The selection of GSP is now explained in the introduction (page 6, lines 132-134).

**Comment 6: There needs to be more explanation of why a segmentation model was chosen over a classification model. How do the authors foresee a segmentation model being used in screening/diagnosis?**

**Response:** We agree that this is an important point. An explanation of why a segmentation model is applied is now added to the introduction (page 6, lines 127-132).

**Comment 7: There are multiple mentions of integration into smartphones/mobile phones. L78-82 suggest that this is the only potential application of this work. However, the types of smartphones that are available and affordable in endemic low-resource settings may not always have the magnification and imaging capabilities to detect GSP lesions. I would encourage the authors to consider that there may be other ways of integrating this technology.**

**Response**: Other methods of model integration have now been mentioned in the introduction on page 6 in lines 136-138.

**Comment 8: What steps were taken to avoid overfitting? As this is a relatively small dataset, some comments on how the risk of overfitting were addressed would be appropriate. The relatively small testing set (n = 87) should also be addressed when discussing the results.**

**Response**: We agree that this should be stated more clearly. On page 12 in lines 262-264, mentions of data augmentation for better model generalizability have been added. In addition, the early-stopping method has been stated to help combat over-fitting on line 265-267. Additionally, mentions of the limited dataset size is stated in the discussion section on page 15 line 330.

**Comment 9: More information on the types of lesions seen in the dataset is warranted. The abstract states that all 583 cervical images exhibited FGS-associated lesions. Please could the authors clarify whether this was any lesion type or GSP specifically? How many images had other lesion types and did the model ever segment these lesions instead?**

**Response**: We appreciate this observation. The 583 cervical images all had GSP lesions present, and this is now stated in the abstract on page 2 line 28.

**Comment 10: More information is needed on the level of imbalance in the dataset. GSP generally only take up a tiny percentage of the overall ROI. This is important for assessing class imbalance and for understanding the use of a focal loss function (and the degree of alpha and gamma used).**

**Response**: Thank you for this comment. Additional explanation about the dataset imbalance and it’s relation to parameter selection has been added to the training parameters section on page 12 lines 260-263 and lines 265-266.

**Comment 11: I cannot see any mention of ethics in this paper. It may fall under the larger study by Arenholt et al. but this needs to be clarified. Some information on how the images were handled (including data security etc) would also be beneficial.**

**Response**: We do agree that an ethics declaration is important and initially it was thought to be covered by the study from which the dataset was obtained. Now an ethics statement referring to the other study, along with the RCT identifier and ethics approval authorization number, has been stated following the dataset section (pages 7-8, lines 162-172). Additionally, a mention of how the images were stored is stated on page 7 line 158.

**Reviewer Specific Points:**

**Comment 12: Abstract: Please could the authors clarify what they mean by non-invasive? Even with a smartphone, the procedure would still require a speculum exam, which many women consider highly invasive. This is also mentioned in L296 and in the conclusion. Visual diagnostics, by their nature, will always be invasive.**

**Response**: Thank you for your comment. The term non-invasive was used earlier as in no mucosa was ruptured however, as reviewer mentioned, visual inspection is also, considered invasive and therefore the term “non-invasive” is no longer used in the paper.

**Comment 13: Abstract: The use of the term “distinct mucosal lesions” would suggest that they are distinctive from other cervical mucosal changes, which is often not the case. Suggest rewording. Mention of ‘confounding cervical diseases’ (diseases that appear visually similar to FGS) would also be beneficial.**

**Response**: On page 2 line 23 it is now reworded to avoid the term “distinct”. Additionally, detail on confounding cervical lesions is left out of the abstract, however, added in the introduction on page 4 lines 87-89

**Comment 13: L15-17: Diagnostic capacity is also constrained by the high levels of subjectivity. I would suggest adding this into your introduction, as reducing subjectivity is a key factor in the benefits of computer vision.**

**Response**: Additional background information about the subjectivity has been added to the introduction on page 4 and 5 on lines 85-94.

**Comment 14: L27: Sigve’s work is not recent. This paper was published ten years ago. Since then, there have been significant advancements computer vision (and FGS research in general).**

**Response**: The term recent has been discarded and more sources on the status of FGS diagnosis have been added throughout the introduction.

**Comment 15: L69-76: Again, I would be hesitant to use the term “visually distinct”. There is limited evidence that GSP could be considered pathognomonic (e.g. Kjetland et al.,2005) but in another paper of hers that year there was no association with eggs found in biopsy etc.**

**Response**: Since this is an ambiguous way of describing GSP and indeed it might not be considered visually distinct, on page 6 lines 127-134, an explanation of why a segmentation model is used as well as why GSP is selected has been provided.

**Comment 16: L83-108: Some more detail on the imaging, annotation and classification process is needed. These details are essential when considering how successfully a model may be able to perform and generalise.**

**Response**: Additional information about the imaging procedure has been added on page 7 on lines 147-154. Additional information about the reviewers and their experience has been added on page 8 in lines 175-178. Reference to study where annotations were performed is now explicitly stated on page 8 in line 182.

**Comment 17: L91: Some brief details on the ‘standardized manner’ would be beneficial.**

**Response**: Details of the imaging procedure have been added on page 7 on lines 147-154

**Comment 18: L96: Please could the authors comment on how many images were discarded due to ‘inadequate image quality? These were separate from the 7 lost during preprocessing? What was considered poor quality (e.g. just blurring? ROI not in view? Discharge in view?)?**

**Response**: Specific explanation on the loss of images in the original study on page 7 on lines 158-161. These image losses are separate from the losses during preprocessing which is explained on page 9 lines 199-202

**Comment 19: L99-108: Please could the authors comment on the number of reviewers, their qualifications and training to label images? Or specifically refer the reader to the relevant paper? These sorts of details are useful when determining the accuracy of the ground truth annotations.**

**Response**: The number of annotators and their qualifications have now been stated and are followed by references to specific studies with their involvement in the annotation process. This has been added on page 8 lines 175-178.

**Comment 20: L98-108: How was the region of interest defined? If your model only focuses on the cervix, and not the surrounding tissue, then this will need to be specified.**

**Response**: We agree that this is an important factor for the model performance. A section on ROI handling has now been on page 10 in lines 221-227. ROI handling is also mentioned on page 8 lines 179-180.

**Comment 21: L119-153: The section detailing the removal of SRs has a large amount of detail compared to other methods sections that may be more relevant to the objective of the paper (and of more interest to a PLOSNTD audience). Much of the detail could be added to a supplementary file to free up space.**

**Response**: The bulk of the specular reflection methodology is now moved into supporting information on page 19-20. In the article the mention of specular reflection removal is still stated however, now it is more focused on the outcome and reference to the supporting information has been added (pages 9-10, lines 206-215)

**Comment 22: L126-127 &135-136: These lines are almost identical. Suggest rewording or removing one for brevity.**

**Response**: The first lines are kept, and the repeat mention has been removed, and section is reworded for better flow pages 19 and 20, lines 433-437

**Comment 23: L151-152: Some more information about the kinds of anomalies and outliers would be useful.**

**Response**: The reduction of images from 590 to 583 resulted from the preprocessing. This is now explained on page 9 in lines 199-202.

**Comment 24: L166-167: The author could provide more detail on the type of hardware used so the reader could appreciate the limitations fully. Information on how data was secured would also be appropriate.**

**Response**: The hardware used is now added on page 11 in lines 243-247.

**Comment 25: L169-170: Your data was longitudinal, with multiple images per woman. Please can the authors comment on how they ensured that images from one woman were not used across both training and testing datasets, as this may create an uplift in model performance. Furthermore, do the authors foresee any risk of bias by having multiple timepoints per woman? Perhaps a comment (and the small amount of evidence we have on lesion progression) on the likelihood of lesions changing over the study period might be warranted?**

**Response**: We thank the reviewer for this comment. This is a valid point and revealed a limitation in the previous experiment design. New models were trained with a correct datasplit at patient-level. Hyperparameters from the previous experiment were used due to time constraints. The new experiment did a 5-fold cross-validation with different patients held out for validation. The test set remained the same. Results now reflect these new models. The datasplit is now defined accurately on page 11-12 in lines 249-256

**Comment 26: L238-239: The mention of visual variability and subtlety here is good. However, think should be mentioned earlier, before the discussion.**

**Response**: The mention of visual variability and subjectivity is now mentioned in the introduction as well on page 4 and 5 in lines 89-94

**Comment 27: L267-284: The model has not been run using any external datasets (understandably), which needs to be mentioned. Furthermore, it does not appear that the model has been run on images that do not have FGS lesions? Without negative controls, how do we know that the model won’t segment ‘GSP-like’ areas in FGS-negative images?**

**Response**: Thank you, we agree that this should be discussed more clearly. A paragraph discussing the limitation of not testing on an external dataset and the mention of no negative controls has now been added to the limitations on page 16 and 17 in lines 362-370
